# Peripheral ulcerative keratitis as presenting manifestation of systemic microscopic polyangiitis: a case report

**DOI:** 10.5935/0004-2749.20220001

**Published:** 2022

**Authors:** David Díaz-Valle, Blanca Benito-Pascual, Rosalía Méndez-Fernández, Pedro Arriola-Villalobos, Gabriel Arcos-Villegas, Mercedes Molero-Senosiain, Mercedes Velo-Plaza

**Affiliations:** 1 Department of Ophthalmology, Hospital Clinico San Carlos, Madrid, Spain; 2 Department of Ophthalmology, Hospital Jiménez Díaz, Madrid, Spain; 3 Department of Ophthalmology, Hospital Gómez Ulla, Madrid, Spain; 4 Department of Nephrology, Hospital Clinico San Carlos, Madrid, Spain

**Keywords:** Corneal ulcer, Microscopic polyangiitis, Systemic vasculitis, Biological dressings, Autoimmune diseases, Úlcera da córnea, Poliangiite microscópica, Vasculite sistêmica, Curativos biológicos, Doença autoimune

## Abstract

Microscopic polyangiitis is a rare autoimmune disease of unknown etiology, characterized by inflammation and necrosis of blood vessels. It forms a part of the antineutrophil cytoplasmic antibody-associated vasculitides-a heterogeneous group of disorders characterized by vasculitis. It is a systemic disease affecting multiple organs. The patients may present with a wide variety of symptoms. Ocular manifestations may present as its initial clinical symptoms, necessitating a multidisciplinary approach for reducing the morbidity and mortality. Early diagnosis aids in the formulation of appropriate treatment and prevention of further complications. Aggressive treatment, including surgery, is often necessary to limit structural damage and preserve visual function. We present the case of an 82-year-old woman who initially presented with peripheral ulcerative keratitis that led to the diagnosis of microscopic polyangiitis.

## Abstract (pt)

A poliangeíte microscópica é uma doença autoimune rara de etiologia desconhecida, caracterizada por inflamação e necrose dos vasos sanguíneos. Faz parte das vasculites associadas a anticorpos citoplasmáticos antineutrófilos - um grupo heterogêneo de doenças caracterizadas por vasculite. É uma doença sistêmica que afeta vários órgãos. Os pacientes podem apresentar uma grande variedade de sintomas. As manifestações oculares podem apresentar-se como seus sintomas clínicos iniciais, necessitando de abordagem multidisciplinar para redução da morbimortalidade. O diagnóstico precoce ajuda na formulação do tratamento adequado e na prevenção de complicações futuras. O tratamento agressivo, incluindo cirurgia, muitas vezes é necessário para limitar o dano estrutural e preservar a função visual. Apresentamos o caso de uma mulher de 82 anos que inicialmente apresentou ceratite ulcerativa periférica que levou ao diagnóstico de poliangite microscópica.

## INTRODUCTION

Ocular manifestations of antineutrophil cytoplasmic antibodies (ANCA)-related vasculitides such as microscopic polyangiitis (MPA) comprise several forms such as orbital pseudotumors, scleritis, retinitis and keratitis. Peripheral ulcerative keratitis (PUK) is characterized by a crescent-shaped stromal inflammation associated with epithelial defect and progressive loss of corneal stroma leading to corneal thinning. It is often accompanied by inflammation of the adjacent conjunctival, episcleral, and scleral tissues, which in turn may lead to serious complications such as corneal perforation^(1)^. In 2015, Sharma et al classified the severity of inflammation based on the extent and depth of the lesions as mild, if they extended up to two o’clock position, affecting the anterior stroma; moderate, if the extension was from two to four o’clock position, affecting the anterior or mid stroma; and severe, if the whole stoma was affected or perforation occurred^(1)^.

Proper management of this disease requires an accurate diagnosis of the underlying cause. Various systemic infectious and non-infectious diseases, microbial organisms (bacteria, fungi, viruses, etc), and vasculitic autoimmune diseases have been implicated in the pathogenesis of PUK^(2)^.

## CASE REPORT

An 82-year-old woman presented to the Ocular Surface and Inflammation Unit of the Ophthalmology Department of our hospital, with acute pain, redness, and vision loss in both eyes. She had been treated for dry eyes for two months. Her medical history was remarkable for anemia and renal insufficiency.

**Figure 1:**
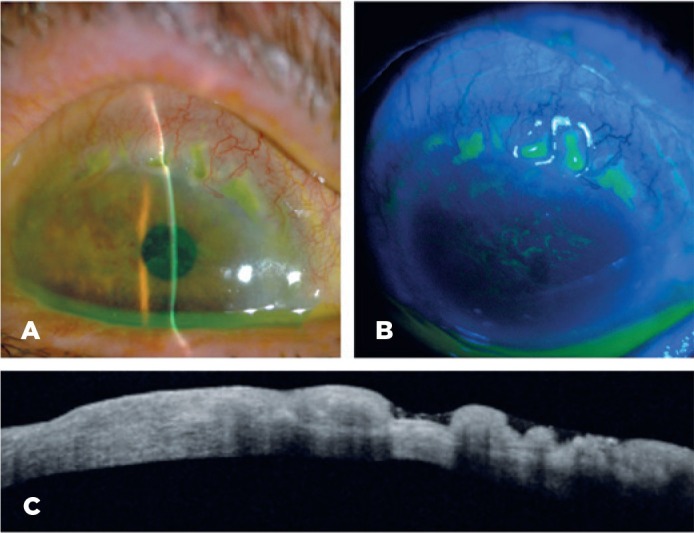
A) A slit-lamp image of the PUK, stromal loss, and inflammation can be seen. B) Fluorescein staining showing epithelial defects in the ulcers. C) Optical coherence tomography of the right affected corneal area showing a severe stromal loss.

Ophthalmologic examination showed a Snellen bestcorrected visual acuity (BCVA) of 20/100 in the right eye (RE) and 20/40 in the left eye (LE). A slit-lamp examination revealed multifocal areas of ulcerative keratitis in the superior peripheral cornea of both eyes (3 o’clock position, from 11 to 2) (Figure 1) with no associated scleritis. Anterior segment optical coherence tomography (AS-OCT) showed a hyper-reflective band with a stromal ulcer with a depth greater than half of the corneal stroma with a corneal thickness of 343 microns (Figure 1C).

A differential diagnosis of infectious diseases was made on initial presentation. Serology tests for herpes, B and C hepatitis, tuberculosis, acquired immune deficiency syndrome, and syphilis were conducted. Corneal scraping culture and chest x-ray were also obtained. All the tests were negative. Due to the possibility of au toimmune disease as the underlying cause, blood tests and renal biopsies were also performed, and nephrologists were also consulted for multidisciplinary management.

The patient was initially treated with eye lubricants (artificial tears and autologous serum eye drops every hour), antibiotics (topical ofloxacin every 6 h, erythromycin ointment twice daily), anticollagenolytics (oral doxycycline 50 mg daily, vitamin C one tablet daily), immunosuppressants (oral deflazacort 30 mg daily and topical cyclosporine 0,05% twice daily), and a regenerating agent (Cacicol^®^, for two cycles).

Hematological tests showed elevated erythrocyte sedimentation rate, and C-reactive protein, creatinine, and complement levels in the serum. Serology test was negative for rheumatoid factor but positive for perinuclear anti-neutrophil cytoplasm antibodies (p-ANCA). Renal biopsy demonstrated segmental glomerulosclerosis, epithelial crescents, collapsed capillaries, tubular atrophy, interstitial fibrosis, mononuclear inflammatory infiltrate, and fibrous thickening of the arterial intima. These results led to the final diagnosis of MPA as they were compatible with p-ANCA vasculitis.

As the initial treatment did not give the desired results, Nephrology prescribed three cycles of intravenous 6-methyl-prednisolone (250 mg) and oral cyclophosphamide (50 mg daily) after MPA was diagnosed. There was some improvement after three weeks of treatment with this regimen. However, due to the persistence of corneal thinning and epithelial defects, we decided to perform amniotic membrane transplantation (AMT) using human fibrin glue (Tissucol^®^). During surgery, the conjunctiva around the lesion was resected to reduce inflammatory mediators and collagenases. Corneal and conjunctival tissue biopsies showed chronic inflammatory changes.

**Figure 2:**
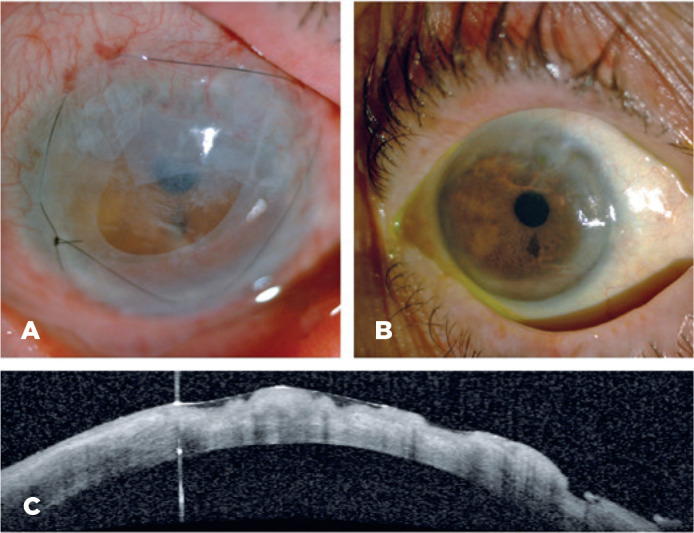
A) First day postoperative after amniotic membrane transplantation. B) A resolution of the ocular injury. C) Tomographic image of the stromal recovery.

A marked improvement in the patient’s symptoms was observed after the surgery, followed by complete resolution a few days later (Figure 2). Due to the progress in the patient’s condition, oral cyclophosphamide was substituted with azathioprine and treatment with topical and systemic steroids was stopped eventually. Four months later, the patient remained stable on topical lubrication and cyclosporine 0.05% twice daily. The BCVA was 20/25 in the RE and 20/32 in the LE, with 100% corneal epithelialization and an almost complete recovery of the stromal thickness (thinnest point 589 microns) (Figure 2C).

## DISCUSSION

Identification of the underlying cause of PUK is crucial, as most of the cases are a result of untreated, inadequately treated, or changes in the treatment of autoimmune systemic diseases. Early identification of the ocular symptoms plays a vital role in improving the patient’s prognosis as ocular involvement precedes systemic involvement in 30% of the patients^(3)^. Laboratory tests and biopsies are important for ruling out infectious diseases^(2)^.

Multidisciplinary management is essential for effective treatment of the condition. In patients with apparently good systemic disease control, PUK requires intense immunosuppressive treatment^(4)^, including aggressive systemic medications along with ocular topical medication. Early and aggressive treatment of PUK leads to a reduction in the ocular morbidity associated with it^(5)^. Refractory or aggressive cases with corneal descemetoceles or perforations require surgical procedures, such as AMT or tectonic lamellar keratoplasty^(3)^.

As MPA rarely manifests in ocular symptoms, a diffe rential diagnostic approach should be adopted for patients presenting with PUK. A multidisciplinary model is fundamental for the successful management of the disease, including the treatment, complications, morbidity, and mortality associated with it.

## References

[1] Sharma N, Sinha G, Shekhar H, Titiyal JS, Agarwal T, Chawla B (2015). Demographic profile, clinical features and outcome of peripheral
ulcerative keratitis: a prospective study. Br J Ophthalmol.

[2] Ladas JG, Mondino BJ. (2000). Systemic disorders associated with peripheral corneal
ulceration. Curr Opin Ophthalmol.

[3] Jia Y, Gao H, Li S, Shi W. (2014). Combined anterior chamber washout, amniotic membrane
transplantation, and topical use of corticosteroids for severe peripheral
ulcerative keratitis. Cornea.

[4] Knox Cartwright NE, Tole DM, Georgoudis P, Cook SD. (2014). Peripheral ulcerative keratitis and corneal melt: a 10-year
single center review with historical comparison. Cornea.

[5] Artifoni M, Rothschild PR, Brézin A, Guillevin L, Puéchal X. (2014). Ocular inflammatory diseases associated with rheumatoid
arthritis. Nat Rev Rheumatol.

